# Gradient-Elution Nanoflow Liquid Chromatography Without a Binary Pump: Smoothed Step Gradients Enable Reproducible, Sensitive, and Low-Cost Separations for Single-Cell Proteomics

**DOI:** 10.1016/j.mcpro.2024.100880

**Published:** 2024-11-12

**Authors:** Kei G.I. Webber, Siqi Huang, Hsien-Jung L. Lin, Tyler L. Hunter, Jeremy Tsang, Dasun Jayatunge, Joshua L. Andersen, Ryan T. Kelly

**Affiliations:** 1 Department of Chemistry and Biochemistry, Brigham Young University, Provo, Utah, USA; 2 Department of Oncological Sciences and Huntsman Cancer Institute, University of Utah School of Medicine, Salt Lake, Utah, USA

**Keywords:** autophagy, HPLC gradient formation, nanoflow liquid chromatography, single-cell proteomics, stepped gradients

## Abstract

Mass spectrometry–based proteome profiling of trace analytes including single cells benefits from liquid chromatography separations operated at low flow rates (*e.g.*, <50 nl/min). However, high-pressure binary pumps needed to achieve such flow rates are not commercially available, and instead require splitting of the gradient flow to achieve low-nanoliter-per-minute flow rates. Gradient flow splitting can waste solvent and lead to flow inconsistencies. To address this, we have developed a method for creating gradients by combining segments of mobile phase having increasing solvent strength together in an open capillary, and then relying on Taylor dispersion to form the desired smooth gradient profile. Our method dramatically reduces costs, as only a single isocratic high-pressure pump is required. Following development of gradient profiles for both 10- and 20-min active gradients, we measured 200 pg injections of HeLa digest using a timsTOF mass spectrometer. Finally, we investigated differences in protein expression between single cells originating from two different colonies of ATG-KO HeLa cells. Thousands of proteins were quantified, and a potential mechanism explaining differential immune responses of these two colonies upon exposure to viral DNA treatment was determined.

## Highlights

• Single-cell proteomics benefits from low-flow liquid chromatography separations.
• Conventional binary pumps require flow splitting to reach low nanoliter-per-minute flow rates.
• A novel system enables highly reproducible nanoflow separations without a binary pump.
• Application to label-free single-cell proteome profiling.

## Graphical Abstract

**Figure:**
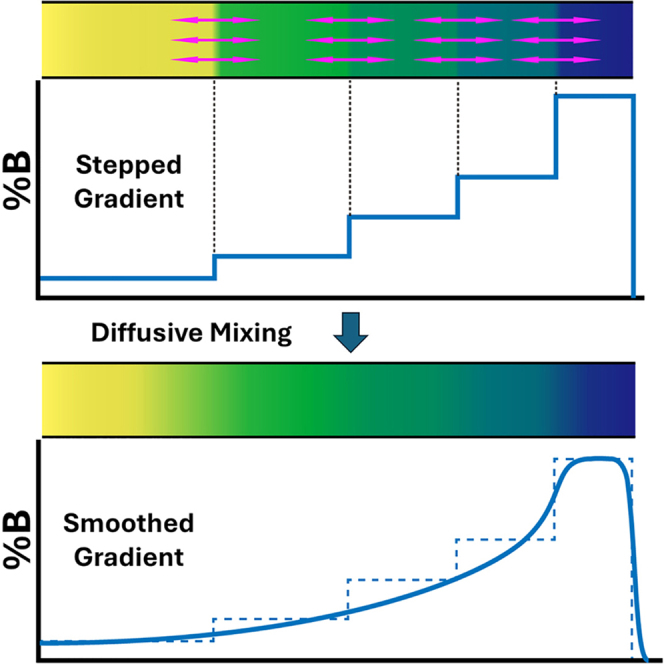

## In Brief

Gradient-elution nanoflow liquid chromatography (nanoLC) systems require flow splitting to reach flow rates below 50 nl/min, which compromise reproducibility. A novel design for nanoflow liquid chromatography replaces costly binary pumps with aspiration of mobile phase plugs having increasing solvent strength into a storage loop. After diffusive mixing, highly reproducible, low-flow gradients are formed. This low-cost nanoLC system is applied to single-cell proteome profiling.

Single-cell proteomics (SCP) has the potential to elucidate mechanisms of disease and detect heterogeneity within cellular populations. However, as there are only picogram amounts of total protein content in single mammalian cells, achieving adequate measurement sensitivity for in-depth SCP is challenging. Efforts to increase sensitivity have led to innovations in all steps of the workflow. For example, one-pot sample preparation using nanowell or microwell plate-based workflows avoid sample losses associated with transfer steps and offline cleanup (1, 2, 3, 4). Other adaptations that benefit SCP include increasing ion transmission in the mass spectrometer and filtering out contaminant species (*e.g.*, with FAIMS or timsTOF) (5, 6, 7, 8), employing a carrier channel for label-based SCP (9, 10), developing novel acquisition methods (11, 12) or employing latest-generation mass spectrometers (5, 6, 13, 14).

SCP also greatly benefits from nanoflow separations due to the resulting increase in ionization efficiency at the electrospray source (15, 16). As such, we routinely operate nanoflow liquid chromatography (nanoLC) separations at flow rates of 20 to 50 nl/min using in-house-packed columns having internal diameters of 20 to 30 μm. However, operating at these low flow rates is very challenging for commercial pumps, as the aqueous and organic mobile phase components (*i*.*e*., solvents A and B) are delivered by separate piston pumps and mixed at high pressure. At early stages of the gradient, flow rates of <1 nl/min may be required of solvent B, which is beyond the capabilities of existing technology. Alternative gradient generation strategies such as low-pressure mixing or quaternary pumps may be employed for analytical flow liquid chromatography (LC) (17, 18), but these have not been commercialized for nanoLC. As such, to achieve ultralow-flow separations, the mobile phase gradient is generally produced at higher flow rates, and a portion of the gradient flow enters the separation column while the balance is diverted to waste. This flow splitting can compromise reproducibility, as slight aberrations in the bulk flow may significantly perturb pressure and flow rates through the nanoflow column. Thus, current methods for creating low-flow gradients are costly, provide limited reproducibility and are limited to binary gradient compositions.

Here, we replace the binary pump with an alternative method for generating mobile phase gradients using a simple selector valve, two switching valves and a single isocratic pump. Discrete mobile phase components are aspirated *via* the selector valve to form a stepped gradient, which is then placed in a gradient formation loop. Due to the dispersive factors of laminar flow and longitudinal diffusion, it is possible to develop smooth mobile phase gradient profiles. While other LC systems have used a single pump to push preformed gradients (13, 19, 20), including at low flows (7, 21), these pumps used traditional means to create those gradients. In addition, stepwise LC gradients have been demonstrated previously (22), and in some cases have been smoothed by dispersive forces to approximate linear gradients (23, 24). However, a binary pump was previously still required to generate discrete mobile phase compositions, and complex valving schemes were utilized to place the components in contact with one another for smoothing. Our system overcomes the cost and minimum flow rate limitations of binary high-pressure pumps by directly creating small-volume gradients with a single syringe pump. We optimized and evaluated the performance of this system using UV absorption spectroscopy and mass spectrometry. We then used the platform for sensitive single-cell proteome profiling. We found that two colonies of the same single-gene knockout cell line followed distinct evolutionary trajectories that resulted in different immune responses to viral DNA infection.

## Experimental Procedures

### Step LC Construction

**Fig. 1:**
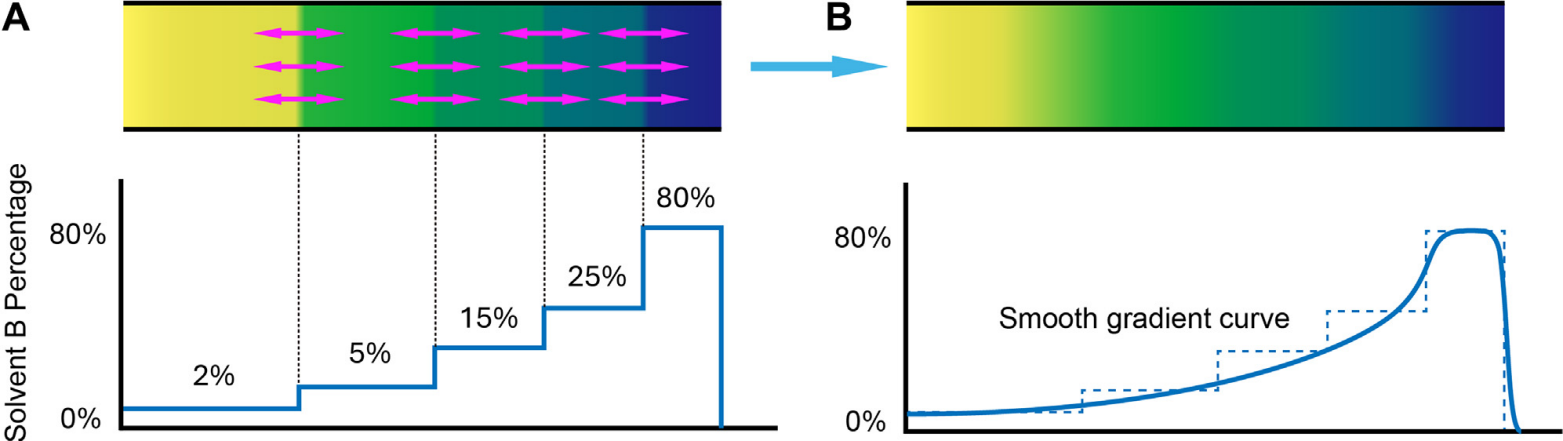
**Step-liquid chromatographyconcept.***A*, a stepped gradient is formed by aspirating plugs of increasing organic solvent composition. *B*, the gradient is then smoothed by a combination of longitudinal diffusion and laminar flow.

**Fig. 2:**
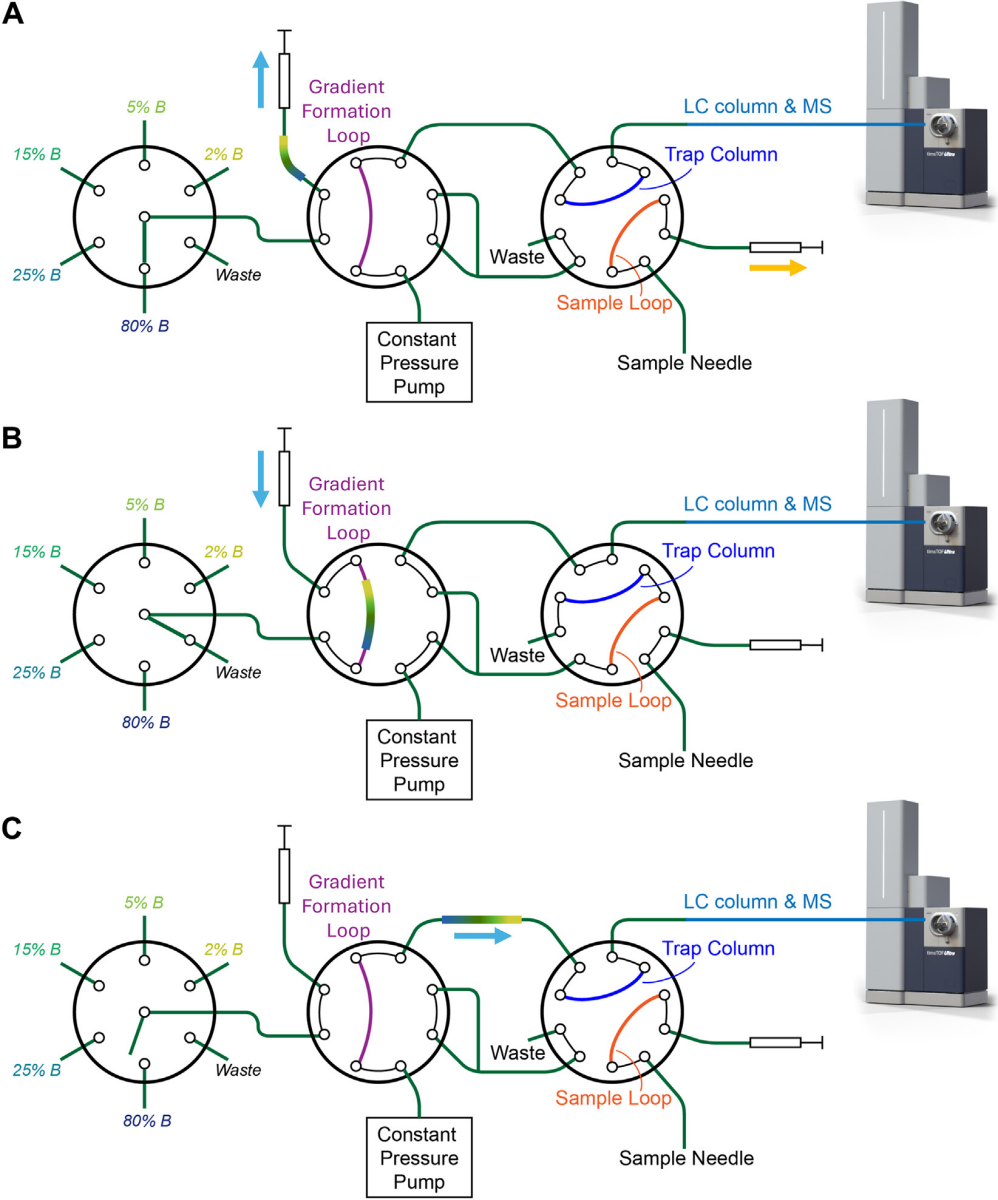
**Step-liquid chromatographydesign and operation.***A*, the gradient (represented by a *multicolored line*) is formed by aspirating solvent plugs through the selector valve (*left*). *B*, the stepped gradient is smoothed as it is loaded and stored in the gradient formation loop, while the split flow loads the sample onto the trap column. *C*, the split flow is closed, and the mobile phase gradient passes through the trap and analytical column, eluting analytes to the MS.

A schematic depiction of gradient formation from discrete mobile phase components is shown in Figure 1. The gradient formation device was constructed as shown in Figure 2. The gradient-formation loop was a 120 cm long × 50 μm internal diameter (i.d.) fused silica capillary (Molex), and connections to the syringe and the selector valve (VICI part #C85NX-4676D) were 75 μm i.d. capillaries. A 10-port Nanovolume two-position valve was purchased from VICI (Part #C72MFSH-4570D) and assembled as previously described (25) to accomplish autosampling and trap-and-elute sample injection (right-hand valve in Fig. 2, *A*–*C*). An 8-port switching valve (VICI, Part #C72MFSH-4578D) was positioned between the selector valve and the autosampling valve (Fig. 2). A syringe pump was constructed from a 10 μl syringe (1701N; Hamilton Company) and a linear stage (X-LSM150A; Zaber) as described previously (1, 26). This was connected to the 8-port valve. The syringe aspirates mobile phase solvent of varying aqueous/organic compositions from vials through the selector valve (left-most valve in Fig. 2, *A*–*C*) and into a connected transfer line (Fig. 2*A*). The autosampler setup was as described previously (27, 28). Sample was aspirated into the sample loop at the same time the mobile phase gradient was formed. As shown in Figure 2*B*, the syringe connected to the 8-port valve positioned the mobile phase gradient into the gradient-formation loop, while a constant pressure pump containing solvent A (0.1% formic acid in water) regenerated the LC column and delivered sample to the trap column in the 10-port valve. As shown in Figure 2*C*, the constant pressure pump delivered the mobile phase gradient to the analytical column through the trap column, while the sample needle was washed. The constant pressure pump was an ASI 541 filled with solvent A and operated at 55 μl/min split with a 100 cm × 30 μm ID capillary (split ratio ∼1000:1) such that the pressure remained at approximately 6000 psi. Note that this split solely serves the purpose of maintaining a constant pressure and has no impact on the gradient. Additionally, the solvent passing through the split column may be recycled to the solvent A reservoir to avoid waste. The selector valve ports 1 to 5 contained 2% B, 5% B, 15% B, 25% B and 80% B, respectively. Port 6 was connected to waste.

### UV Acquisition

All UV absorption data were acquired at 264 nm with the ECD2600 CE UV-Vis Detector (ECOM) through a 75 μm i.d. fused silica capillary that had the polyimide coating removed. The gradient output to the 10-port valve was disconnected such that the UV signal was measured directly after leaving the 8-port valve. The flow rate was reduced to approximately 60 nl/min by adding a 100 cm long × 5.5 μm i.d. capillary after the detection capillary or to 25 nl/min by adding a 200 cm long 5 μm i.d. capillary after the detection capillary. Solvent B was changed to contain 10% acetone in acetonitrile to facilitate UV detection.

### Culture, Isolation, and Preparation of Single-Cell Samples

HeLa cells were cultured in Dulbecco’s modified Eagle’s medium (Gibco, 11965-092) supplemented with 10% fetal bovine serum (Genesee Scientific, 25-514) at 37 °C in a 5% CO2 incubator. Autophagy protein 5 (ATG5) KO HeLa cells (American Type Culture Collection) were generated using single-guide RNA (gRNA) 5′- AAGAGTAAGTTATTTGACGT-3′ against human ATG5 (ENST00000369076.8). CRISPR design tools, available at www.atum.bio and crispr.mit.edu, were used. The gRNAs were cloned into the pSpCas9(BB)-2A-Puro (PX459) plasmid. PX459 was a gift from Dr Feng Zhang (Addgene, 48139). Cells expressing the gRNA constructs were microscopically sorted and isolated into monoclonal cultures under puromycin selection. Knockout efficiency was measured by Western blotting with the following antibodies: ATG5 rabbit monoclonal (Cell Signaling Technology, 12994), actin (Cell Signaling Technology, 4970S). To compare the type I interferon signaling response between different KO clones, cells were treated with 1 μg/ml poly dA:dT for 24 h. Cells were then lysed and immunoblotted for phosphorylated interferon regulatory factor 3 (IRF3) using the phospho-IRF3 (ser386) rabbit monoclonal antibodies (Cell Signaling Technology, 37829).

Single HeLa cells were isolated using the cellenONE (Cellenion). Cells having a maximum elongation factor of two were selected for isolation into LoBind 384-well PCR plates (part # 0030129547, Eppendorf). Five hundred nanoliters of digest solution (2.5 ng/2.5 ng Trypsin/LysC and 0.01% N-dodecyl-β-D-maltoside) were dispensed into each cell-containing well using the Tecan Uno (2). The PCR well plate was then incubated for 1 h at 70 °C. Well plates were centrifuged after each step to drive cells and reagents to the bottom of the wells. To avoid batch effects, cells from both colonies were processed together on the same well plate.

### Gradient Formation and Separation

All gradient plugs were aspirated at 3.0 μl/min into 75 μm i.d. tubing that connected the syringe to the 8-port valve as shown in Figure 2*A*. For 10-min gradients, the step gradient was then immediately placed in the 50-μm-i.d., 2.35-μl gradient formation loop by dispensing from the same syringe at 1.0 μl/min (Fig. 2*B*). Concurrently, the sample was loaded onto the trap column using a split flow from the constant pressure pump as also shown in Figure 2*B*. For the 20-min active separations, the same procedure was followed, but when the gradient components were aspirated from their corresponding reservoirs, they were held in the connecting tubing for 10 min to provide more diffusional mixing prior to placing in the gradient formation loop. For both 10- and 20-min separations, the valve was switched as shown in Figure 2*C* so that the flow from the constant pressure pump drove the stored gradient through the trap and analytical columns to effect separations. Gradient compositions were as follows. For 10-min separations, plug 1 was 300 nl of 2% B, plug 2 was 200 nl of 5% B, plug 3 was 300 nl of 15% B, plug 4 was 200 nl of 25% B, and plug 5 was 600 nl of 80% B. For 20-min gradients, plug 1 was 50 nl of 2% B, plug 2 was 700 nl of 5% B, plug 3 was 50 nl of 25% B, plug 4 was 200 nl of 15% B, plug 5 was 50 nl of 25% B, plug 6 was 200 nl of 15% B, plug 7 was 450 nl of 25% B, and plug 8 was 500 nl of 80% B. The gradient for the 25 nl/min UV-acquisitions was identical to that employed for the 10-min separations.

The analytical column was a 30 μm i.d., 20-cm long fused silica capillary that was home packed with 1.9 μm Dr Maisch C18 media (Part No. r119.aq.0001). The trap column was a 75 μm i.d. capillary home-packed with 5 cm of the same media (12).

### Mass Spectrometry

Samples were analyzed using a timsTOF Ultra mass spectrometer (Bruker Corp) in a randomized, blocked sequence. The LC system was interfaced with a home-etched 10 μm i.d. emitter (29) that was inserted into a CaptiveSpray ESI source. Precursor data were acquired in the range of 100 to 1700 *m/z* and 1.45–0.64 1/K_0_ [V·s/cm^2^] in diaPASEF mode (7). Fifteen data-independent acquisition (DIA) windows spanned 400 to 1000 *m/z* and were acquired with matched trapping and ramp times of 100 ms. Specific window placement is provided in Supplemental Fig. S1. The collision energy was ramped as a function of increasing mobility starting from 20 eV at 0.6 1/K_0_ to 63 eV at 1.6 1/K_0_. No denoising mode was applied for diaPASEF data reduction.

### Data Analysis

UV data were exported from ECOMAC as a text file, and postprocessing and visualization were performed using Python. Calibration of UV absorption data was accomplished by measuring 1.4 μl plugs of each solvent of known %B in triplicate. A piecewise function of the signal-concentration pairs was then used to convert UV signal of measured gradients into %B. The gradients were plotted using the plotly package in python.

Mass spectrometry data were analyzed with the fractionated human spectral library provided by Bruker, which contains 13,103 entries, using DIA-NN software Version 1.8.1 (30). The specified enzyme was trypsin, and no missed cleavages were permitted. Mass tolerance for fragment ions was dynamically selected for each file by DIA-NN. Oxidation and acetylation were set as variable modifications, and 1% false discovery rate (FDR) was selected for both precursors and proteins. Postprocessing was performed using Python and R. All analyses employing 10-min gradients were processed together, but separately from the analyses that employed 20-min gradients. For all samples, common contaminants were removed from the report.pg_matrix.tsv file before counting identified precursors and proteins, and error bars represented ± 1 SD. Only proteins with a nonzero abundance value were considered as identified. Peak retention time shifts were calculated as SDs of the retention times in the report.tsv output file from DIA-NN.

For the volcano plot and principal component analysis (PCA) plot of single cells, the report.pg_matrix.tsv file was first filtered for the relevant files (*i.e.*, bulk samples and 7 failed injections with no identifications were excluded), and common contaminants as well as proteins with all missing values were removed. The data were then normalized separately for each cell line using the SCnorm package in R and exported as a TSV file. For volcano plots, the *p* values were corrected using Benjamini–Hochberg FDR. For PCA plots, the same TSV data were log2-transformed before median normalization, then missing values were imputed using K nearest neighbors with a k = 5, and input into the sklearn PCA function. All plots were generated using the plotly package.

### Experimental Design and Statistical Rationale

**Fig. 3:**
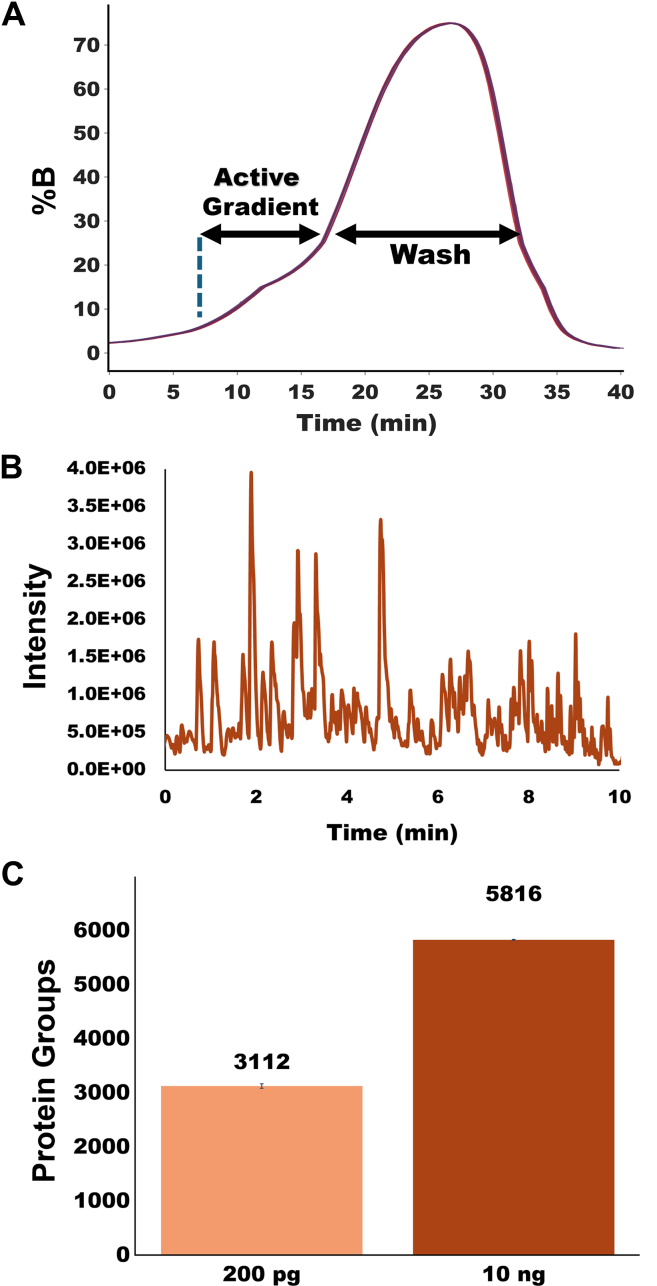
**Ten-min active gradients.***A*, gradient profile for 10 overlaid gradients as measured by UV absorption spectroscopy. *B*, base peak chromatogram of the active gradient for analysis of 10 ng HeLa digest, with time = 0 set when peptides begin to elute. *C*, number of identified protein groups for analysis of 200 pg and 10 ng aliquots of HeLa digest.

**Fig. 4:**
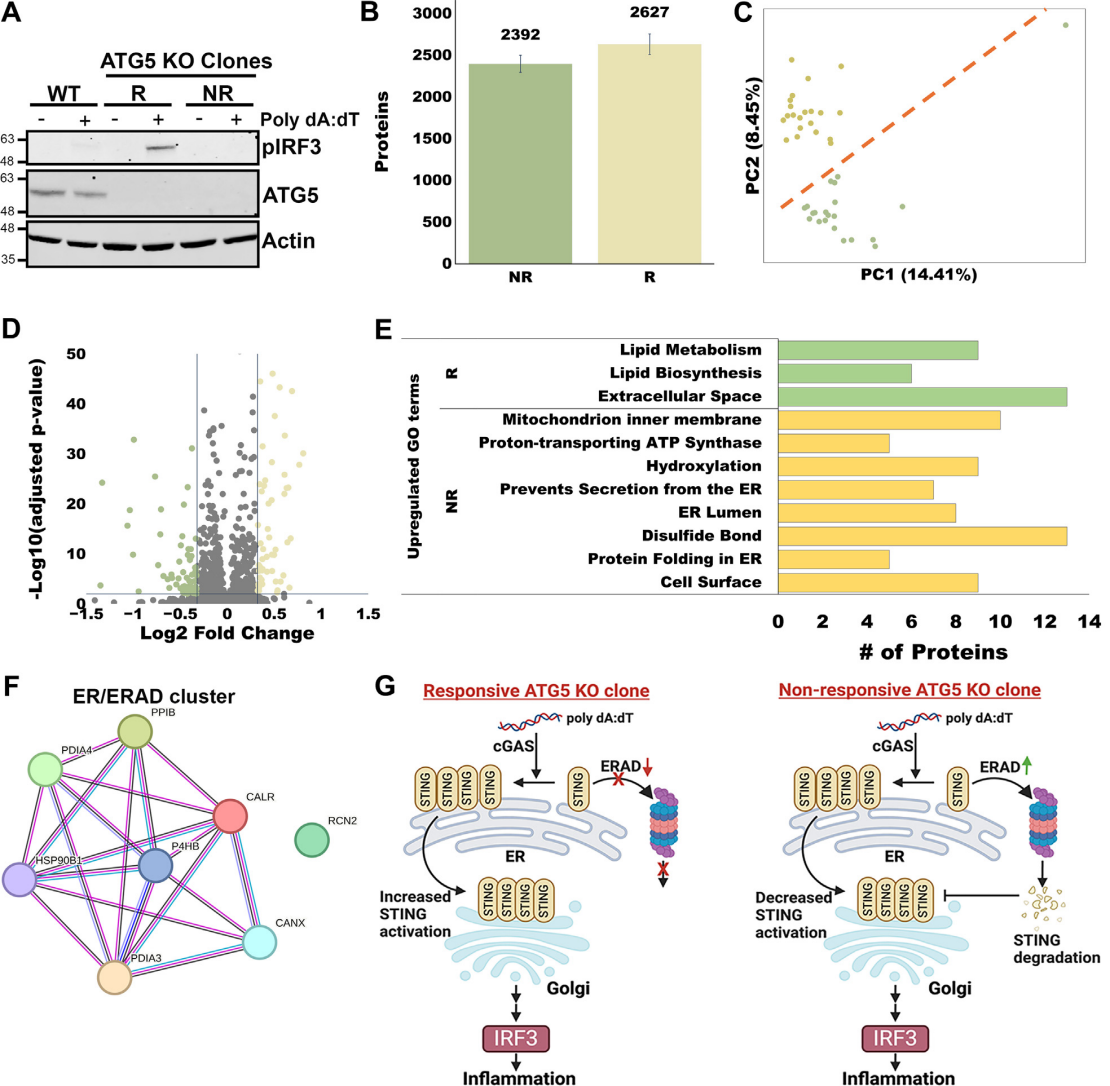
**Analysis of two colonies of ATG-KO HeLa cells analyzed with 20-min active gradients.***A*, Western blot of HeLa WT, ATG5 KO responsive clone (R), and ATG5 KO nonresponsive clone (NR), treated with or without 1 μg/ml poly dA:dT for 24 h. *B*, protein identifications analyzed with match between runs using a 10-ng bulk HeLa digest library. *C*, PCA plot, color coded as *green* (R) and *yellow* (NR). The *red line* shows separation between the two colonies. *D*, volcano plot of differentially expressed proteins with an alpha of 0.01 and a minimum fold change of 1.25. *E*, enriched gene ontology terms for each population. *F*, STRING analysis showing a cluster of ER/ERAD-associated proteins that are upregulated in the NR clone. *G*, a model to explain how autophagy-adapted cancer cells can suppress STING- and IRF3-mediated inflammatory signaling by increasing expression of ERAD-associated proteins. ATG5, autophagy protein 5; ERAD, ER-associated degradation; IRF3, interferon regulatory factor 3; PCA, principal component analysis; STING, stimulator of interferon genes.

Sample sizes were 10 and 1 for Figure 3, *A* and *B*. For Figure 3*C*, sample sizes were 10 (200 pg) and 5 (10 ng). For Figure 4, *B*–*G*, there were 21 of each cell type included. Seven failed injections due to improper autosampler positioning were excluded. Wilcoxon tests were used in the place of *t* tests, as a normal distribution of protein abundances could not be assumed due to unpredictable cellular heterogeneity. For SI Supplemental Fig. S1, *A* and *B*, there were 10 and 1 replicates, respectively. For S1C, there were 8 and 4 replicates, respectively. Supplemental Fig. S3 has 45 replicates.

## Results

We have developed an alternative system that creates gradients by diffusing together plugs of increasing solvent strength and then pushing this gradient through the analytical column with a single isocratic, constant-pressure pump. This pump is far less complex and therefore much more affordable than a typical binary pump, and it can reach lower flow rates without splitting the gradient flow. We anticipated that this technology would allow for more affordable and reproducible gradient formation at low nanoliter-per-minute flow rates. To evaluate system performance, we first created 10- and 20-min active gradients and measured them using both UV absorption and MS. Using UV absorption, we were able to observe that both gradient lengths formed by step-LC were consistent and reproducible. Indeed, Figure 3*A* shows ten superimposed gradient profiles that are virtually indistinguishable from one another. The highly reproducible gradients were confirmed with MS-based proteome profiling. Across eight replicate analyses of 0.2 ng HeLa digest, the median unnormalized SD of retention time for common peptides across eight replicates was just 3.7 s for the 10-min gradients. This relative SD of just 0.6% of the total peptide elution window compares very favorably with a prior study of four different commercial nanoLC systems, which were found to have relative SD between 0.7% and 2.2%, even when employing longer gradients and higher flow rates (31). While the active elution portion of the gradients is not perfectly linear, the resulting chromatograms and the constant rates of peptide identification show that these pseudolinear smoothed gradients are of sufficient quality for 10-min active-gradient separations (Fig. 3*B*). Similarly reproducible gradient profiles and high-peak-capacity separations were obtained for 20-min active gradients as shown in Supplemental Fig. S2, and a representative base peak chromatogram for the full 20-min gradient including overhead steps is shown in Supplemental Fig. S3, with active peptide elution beginning at 15 min.

Although we were somewhat limited in the gradient profiles that could be formed due to the finite number of solvent compositions in each of the five solvent ports, it was possible to approximate intermediate concentrations by alternating between reservoirs of different solvent strength with sufficiently small plug sizes, which then diffused together. For the 20-min gradient, we thus approximated a 17% B by placing a 50 nl 25% B plug in between two 200 nl 15% B plugs, yielding smooth gradients and a high-quality separation (Supplemental Fig. S2, *A* and *B*). As such, we demonstrated that pseudolinear gradients of different lengths can be formed with the same system. Additionally, we were able to form consistent gradients at 25 nl/min across 45 runs (Supplemental Fig. S4). Though the variation was slightly greater, this demonstrates that the system is capable of low-flow gradient formation.

Next, 200 pg injections of commercial HeLa digest yielded an average of 2890 and 3049 protein identifications for 10- and 20-min active gradients, respectively, with median CVs of 10.5% and 12.3% (Fig. 3*C* and Supplemental Fig. S2*C*). While it is difficult to compare performance on a different mass spectrometer and with different column dimensions, our system performs similarly to the commercially available liquid chromatography systems used in previous published work by us using 20-min active gradients (12) and by others using 15-min active gradients (14).

We used the smoothed step gradient system to determine proteome-level differences between clonal populations of HeLa cells that have adapted different responses to the loss of a critical autophagy gene. Autophagy is a protein/organelle recycling process that can promote cancer cell growth, as cancer cells rely on high levels of autophagy to suppress proinflammatory signaling and evade antitumor immunity (32, 33, 34, 35). To generate these autophagy-deficient clonal lines, we treated a single population of HeLa cells with CRISPR/Cas9 to delete ATG5, an essential autophagy gene, then sorted single cells into dishes and collected the surviving clonal populations. As expected, due to the loss of autophagy, most of the clones showed increased sensitivity to proinflammatory signaling triggered by exogenous AT-rich DNA (dA:dT), which mimics viral DNA. However, as shown in Figure 4*A*, one clone showed a complete loss of proinflammatory response to dA:dT as measured by phosphorylation of the proinflammatory transcription factor IRF3. Therefore, we applied our SCP approach to this clone, termed “nonresponder” (NR), and compared it to one of the “responder” (R) clones that showed the expected increase in dA:dT-triggered inflammatory signaling.

Single cells from the NR and R clonal populations yielded an average of 2627 and 2392 identified proteins when using 20-min gradients (Fig. 4*B*). This level of single-cell proteome coverage agrees with expectations based on a recent study of Ctortecka *et al*. (14), which also used the timsTOF Ultra. They identified 3500 proteins per cell with a 30-min active gradient, and 1200 proteins per cell with a 15-min active gradient. Cells from the two colonies analyzed in the present study were separated using PCA (Fig. 4*C*). Additionally, after filtering for an FDR-adjusted *p* value ≤0.01 and a 25% or greater fold change in protein abundance, we found over 100 differentially expressed proteins (Fig. 4*D*). Notably, there is a significant enrichment in upregulated proteins corresponding to gene ontology terms related to lipid metabolism in R and mitochondria in NR (Fig. 4*E*). Interestingly, the NR clones showed an upregulation of proteins associated with an endoplasmic reticulum-associated degradation pathway that suppresses dA:dT-mediated IRF3 signaling by degrading the stimulator of interferon genes (STING) protein (Fig. 4, *F* and *G*) (36, 37). While STING was not detected in any of our library or sample runs, these data suggest a model in which cancer cells can sporadically adapt to loss of autophagy by suppressing STING-driven inflammatory signaling, perhaps through epigenetic or posttranslational regulation of endoplasmic reticulum-associated degradation components.

## Discussion and Conclusion

Our results show that smoothed step-gradient LC is capable of performing gradient separations similar to those using conventional binary pumps, while avoiding the need for splitting of the mobile phase gradient for operation at ∼25 to 60 nl/min. While other pumps have used a single pump to push preformed gradients at low flows, these pumps used binary pumps to create those gradients (7, 13, 20, 21). Step-LC generates gradients with a syringe pump and selector valve at a much lower cost. Likewise, unlike a traditional binary pump, Step-LC can accommodate additional solvents without adding another pump, which might otherwise further compromise reproducibility at low flow rates. This opens the possibility of utilizing ternary gradients at low flow rates, allowing for sensitive analysis of multiple classes of biomolecules (*e.g.*, peptides and lipids).

The described system has proven capable of elucidating slight differences in protein expression between populations of the same cell line that resulted from distinct evolutionary adaptation in response to autophagy inhibition. The system for reproducible gradient generation utilized a novel method and was constructed for roughly one 10th of the cost of commercial nanoLC systems. One limitation of the system is that the duty cycle is currently <50% such that measurement throughput was limited to 27 to 34 samples per day. However, for this proof-of-concept system, the throughput was limited by the need for sample loading onto the solid-phase extraction column and by using a standard single column system at low flow rates and not by the creation of gradients, which took only a few minutes. Such limitations could be overcome by changing the setup of the autosampler to include two analytical columns that simultaneously collect samples, load them onto the solid-phase extraction column, analyze them, and then regenerate the column, without changing the method for gradient formation (27). Alternatively, it would still be cost effective to create an entire parallel system for another ∼$10,000 USD, as the step-LC system is relatively inexpensive to construct, comprising only a selector valve, a syringe pump, a high-pressure syringe pump, and a two-position valve with a gradient formation loop. Nonetheless, our system gave us insight into mechanisms whereby ATG-KO HeLa cells can eliminate responsiveness to viral DNA by deactivating the IRF3-mediated inflammation response pathway through STING degradation and is expected to benefit a number of other applications that require low-flow separations.

## Data Availability

All postprocessing code can be found at https://github.com/RTKlab-BYU/StepLC-Data. The mass spectrometry proteomics data have been deposited to the ProteomeXchange Consortium *via* the PRIDE (38) partner repository with the dataset identifier PXD052416.

Gradient-elution nanoLC systems require flow splitting to reach flow rates below 50 nl/min, which compromises reproducibility. A novel design for nanoLC replaces costly binary pumps with aspiration of mobile phase plugs having increasing solvent strength into a storage loop. After diffusive mixing, highly reproducible, low-flow gradients are formed. This low-cost nanoLC system is applied to single-cell proteome profiling.

## Supplemental Data

This article contains supplemental data.
