# Supplementary material for: Gradient-Elution Nanoflow Liquid Chromatography Without a Binary Pump: Smoothed Step Gradients Enable Reproducible, Sensitive, and Low-Cost Separations for Single-Cell Proteomics
Source: Mol Cell Proteomics. 2024 Nov 12;23(12):100880. doi: 10.1016/j.mcpro.2024.100880 (PMC11667035; doi:10.1016/j.mcpro.2024.100880)
Supplement: Supplementary Information [file mmc1.pdf]

Supporting Information for:

Gradient-Elution Nanoflow Liquid Chromatography without a Binary Pump: Smoothed Step Gradients Enable Reproducible, Sensitive, and Low-Cost Separations for Single-Cell Proteomics

Author List

Kei G. I. Webber,[1,†] Siqi Huang,[1,†] Hsien-Jung L. Lin,[1] Tyler L. Hunter,[1] Jeremy Tsang,[2] Dasun Jayatunge,[2] Joshua L. Andersen,[2] Ryan T. Kelly[1*]

[1]Department of Chemistry and Biochemistry, Brigham Young University, Provo, Utah

[2]Department of Oncological Sciences and Huntsman Cancer Institute, University of Utah School of Medicine, Salt Lake City, Utah

† Equal contribution from both authors

* Corresponding Author

Figure S1. Mass Spectrometry Settings for timsTOF-Ultra

Figure S2. Step-LC 20-Minute gradient profiles and chromatograms

Figure S3. Representative full base peak chromatogram for 20-min active elution, including time prior to and following peptide elution, which takes place from 15–35 min.

Figure S4. Forty-five overlaid gradient profiles as measured by UV absorption spectroscopy when the system operated at 25 nL/min.

Supporting Information

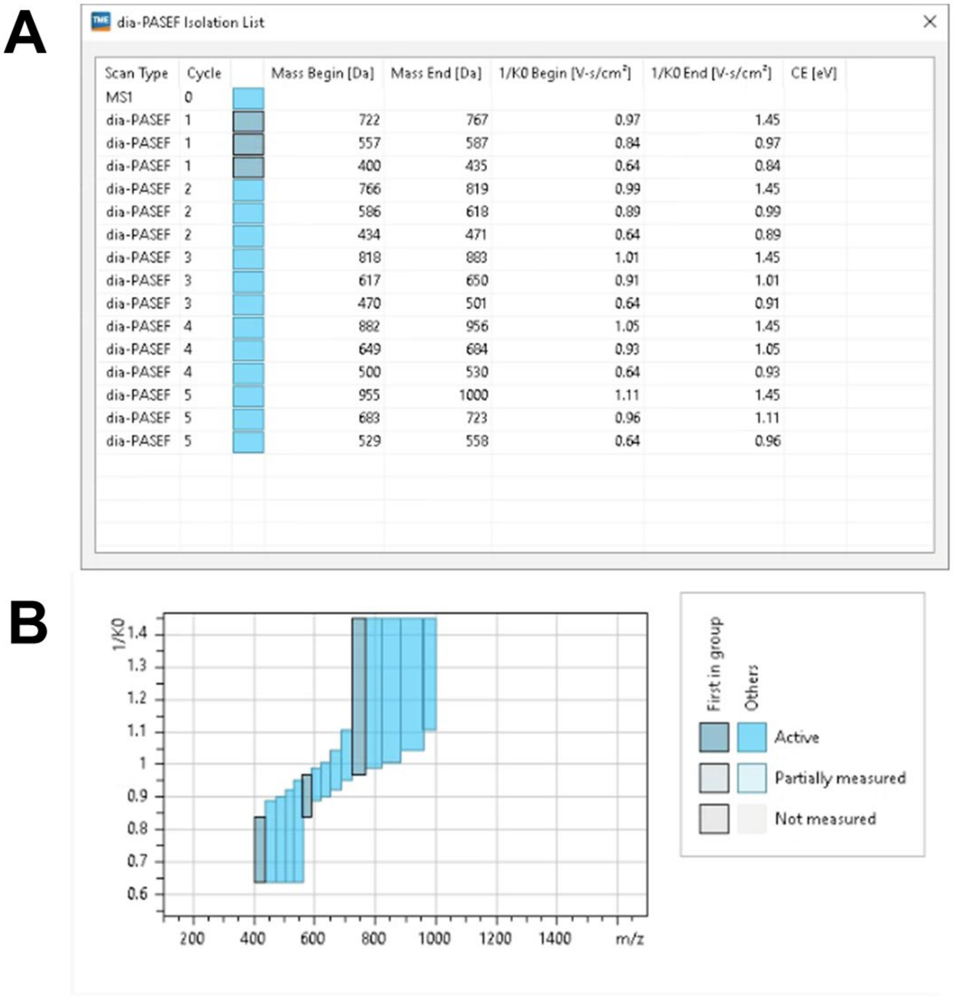

A

dia-PASEF Isolation List

Scan Type	Cycle		Mass Begin [Da]	Mass End [Da]	1/K0 Begin [V-s/cm²]	1/K0 End [V-s/cm²]	CE [eV]
MS1	0						
dia-PASEF	1		722	767	0.97	1.45	
dia-PASEF	1		557	587	0.84	0.97	
dia-PASEF	1		400	435	0.64	0.84	
dia-PASEF	2		766	819	0.99	1.45	
dia-PASEF	2		586	618	0.89	0.99	
dia-PASEF	2		434	471	0.64	0.89	
dia-PASEF	3		818	883	1.01	1.45	
dia-PASEF	3		617	650	0.91	1.01	
dia-PASEF	3		470	501	0.64	0.91	
dia-PASEF	4		882	956	1.05	1.45	
dia-PASEF	4		649	684	0.93	1.05	
dia-PASEF	4		500	530	0.64	0.93	
dia-PASEF	5		955	1000	1.11	1.45	
dia-PASEF	5		683	723	0.96	1.11	
dia-PASEF	5		529	558	0.64	0.96	

Figure S1. Mass Spectrometry Settings for DIA PASEF using the Bruker timsTOF Ultra in (A) table form (B) plotted as $1/K_0$ vs. *m/z*.

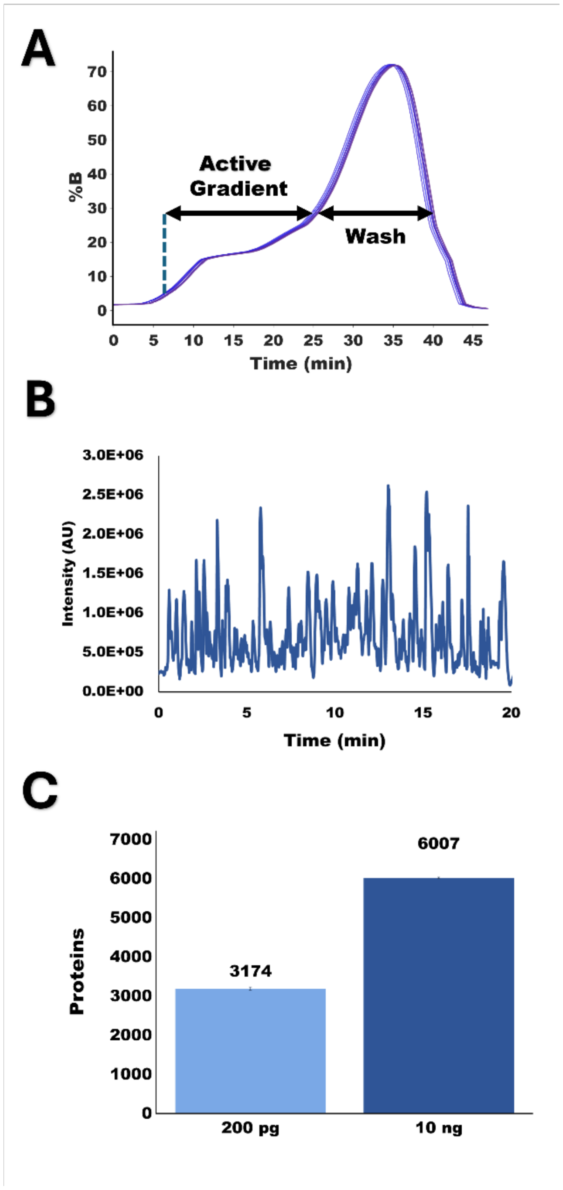

Figure S2. Twenty-minute active gradients. (A) Gradient profile for ten overlaid gradients as measured by UV absorption spectroscopy. (B) Base peak chromatogram of the active gradient for analysis of 10 ng HeLa digest. (C) Number of identified protein groups for analysis of 200 pg and 10 ng aliquots of HeLa digest.

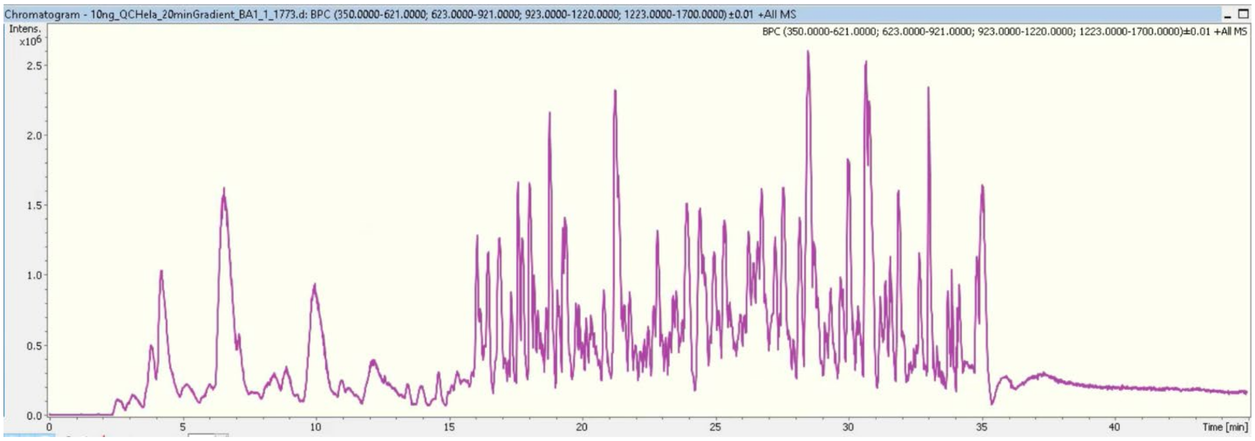

Figure S3. Representative base-peak chromatogram for a 20-min active gradient, which shows the entire chromatographic cycle including column regeneration and washing following peptide elution, which takes place from 15–35 min.

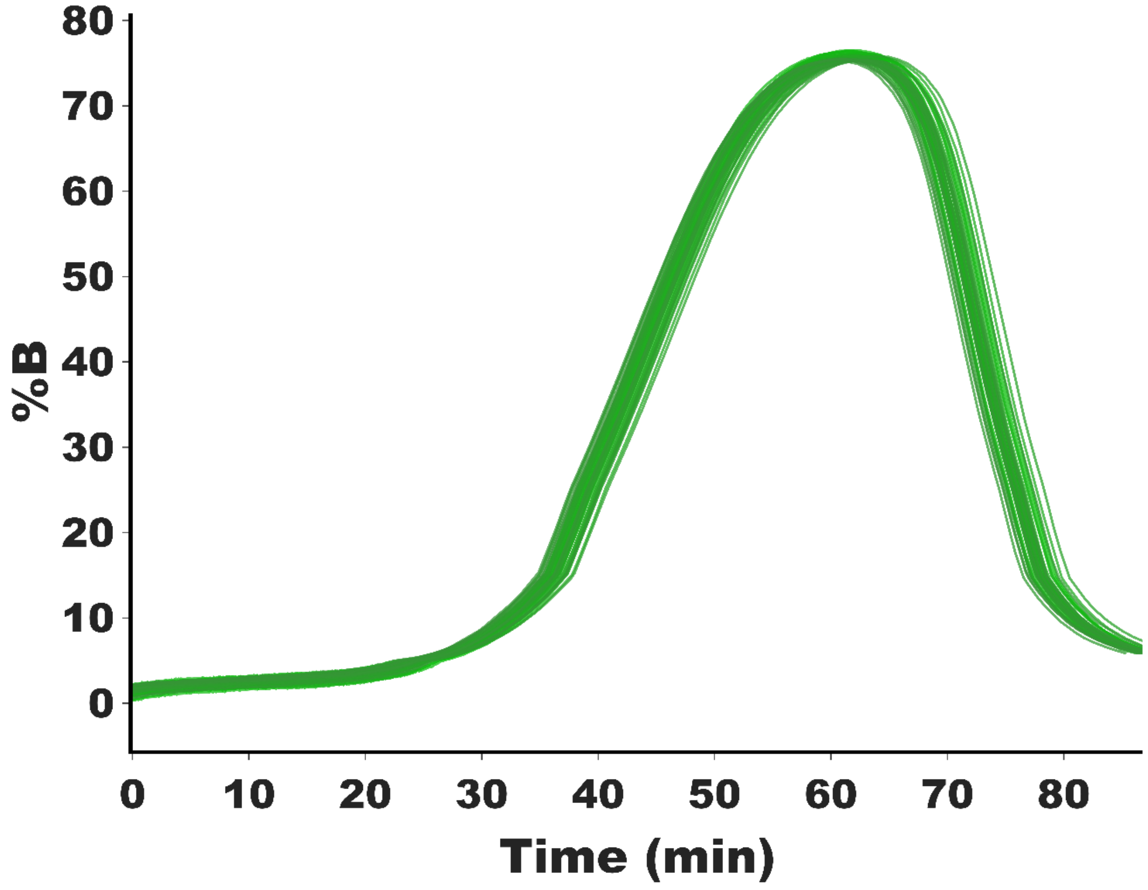

Figure S4. UV profile of 45 replicate 25 nL/min gradients.
